# Transcriptome Analysis of Ovarian and Uterine Clear Cell Malignancies

**DOI:** 10.3389/fonc.2020.598579

**Published:** 2020-12-22

**Authors:** Jill Alldredge, Leslie Randall, Gabriela De Robles, Anshu Agrawal, Dan Mercola, Marisa Liu, Pavneet Randhawa, Robert Edwards, Michael McClelland, Farah Rahmatpanah

**Affiliations:** ^1^ Department of Obstetrics and Gynecology, University of Colorado, Aurora, CO, United States; ^2^ Department of Obstetrics and Gynecology, Virginia Commonwealth University, Richmond, VA, United States; ^3^ Department of Pathology and Laboratory Medicine, University of California, Irvine, CA, United States; ^4^ Department of Immunology, University of California, Irvine, CA, United States; ^5^ Department of Obstetrics and Gynecology, University of California, Irvine, CA, United States; ^6^ Department of Microbiology and Molecular Genetics, University of California, Irvine, CA, United States

**Keywords:** ovarian clear cell cancer, uterine clear cell carcinoma, transcriptome (RNA-seq), PD-L1, immune microenvironment

## Abstract

**Purpose:**

Ovarian and uterine clear cell carcinomas (CCCs) are rare but associated with poor prognosis. This study explored RNA transcription patterns characteristic of these tumors.

**Experimental Design:**

RNA sequencing (RNA-seq) of 11 ovarian CCCs and five uterine CCCs was performed and compared to publicly available data from high grade serous ovarian cancers (HGSOCs). Ingenuity Pathway Analyses were performed. CIBERSORT analyses estimated relative fractions of 22 immune cell types in each RNA-seq sample. Sequencing data was correlated with PD-L1 immunohistochemical expression.

**Results:**

RNA-seq revealed 1,613 downregulated and 1,212 upregulated genes (corrected p < 0.05, |FC |≥10) in ovarian CCC *versus* HGSOC. Two subgroups were identified in the ovarian CCC, characterized by ethnicity and expression differences in *ARID1A*. There were 3,252 differentially expressed genes between PD-L1+/− ovarian CCCs, revealing immune response, cell death, and DNA repair networks, negatively correlated with PD-L1 expression, whereas cellular proliferation networks positively correlated with expression. In clear cell ovarian *versus* clear cell uterine cancer, 1,607 genes were significantly upregulated, and 109 genes were significantly downregulated (corrected p < 0.05, |FC|≥10). Comparative pathway analysis of late and early stage ovarian CCCs revealed unique metabolic and *PTEN* pathways, whereas uterine CCCs had unique Wnt/Ca+, estrogen receptor, and CCR5 signaling. CIBERSORT analysis revealed that activated mast cells and regulatory T cell populations were relatively enriched in uterine CCCs. The PD-L1+ ovarian CCCs had enriched resting NK cells and memory B cell populations, while PD-L1− had enriched CD8 T-cells, monocytes, eosinophils, and activated dendritic cells.

**Conclusions:**

Unique transcriptional expression profiles distinguish clear cell uterine and ovarian cancers from each other and from other more common histologic subtypes. These insights may aid in devising novel therapeutics.

## Introduction

Ovarian and uterine carcinomas are gynecologic malignancies with significantly associated morbidity and mortality. Clear cell carcinoma (CCC) is a histologic subtype of both ovarian and uterine cancer that demonstrates unique clinical behavior. While other histologies display microarray gene expression patterns unique to their tissue of origin, CCCs show a remarkably similar gene expression pattern in the endometrium, kidney, and ovary (1, 2). Both uterine and ovarian clear cell carcinoma are rare and have early metastasis, high risk of recurrence, and poor prognosis (3). Given that these represent less than 5% of all uterine or ovarian cancers, optimal management strategies are extrapolated from more common histologies and incorporate comprehensive surgical staging and combination cytotoxic chemotherapy (4).

Tumor growth and responsiveness to therapy have foundations in tumor-induced immune suppression, primarily regulated by programmed death-ligand 1 (PD-L1), which promotes a regulatory T cell population and thereby facilitates immune evasion. The immunohistochemical expression of PD-L1 is correlated with poor outcomes in renal CCC (5) and ovarian CCC (6). Additionally, mutations in TP53, *PIK3CA, PRKC1,* and *KRAS* oncogenes as well as *ARID1A* and *PTEN* tumor suppressor genes have been studied as key drivers of ovarian CCCs, revealing therapeutic targets (7–9). Explorations of the genetic and epigenetic pathophysiology of CCCs are limited by the rarity of the tumors. Two studies analyzing DNA from 39 ovarian CCCs or 16 uterine CCCs report on mutational loads, microsatellite stability, and frequency of pathologic somatic mutations (10, 11).

An emerging field of study in tumorigenesis is the study of the tumor microenvironment, which includes the cellular and non-cellular components surrounding a tumor that allow it to acquire attributes of immune evasion, unchecked replication, invasion and metastasis. In renal CCC, breast, lung and colon cancers, immune signatures have been identified in the microenvironment with prognostic implications as well as a potential for precision immunotherapeutic targeting (12–15). This study is the first assessment of the tumor microenvironment of uterine and ovarian CCCs.

The primary aims of this retrospective cohort study were to describe whole-genome RNA sequencing patterns in uterine and ovarian CCC populations and to explore differential gene expression between tissues of different origins, stage, different expression of PD-L1, and between ovarian CCC and the more common high-grade serous histology. This study also explores the immune microenvironment of ovarian and uterine CCCs by evaluating the relative fractions of immune mediator cells in each cancer.

## Materials and Methods

### Patient Selection

Ovarian and uterine tumors were identified in the University of California, Irvine, Tissue Biorepository, that had been collected between 1992 and 2017 for women aged 18 years and over and were reviewed again to ensure they had pure clear cell histology and adequate available paraffin-embedded formalin-fixed tissue for staining. Mixed tumors that had any non-CCC histology were excluded, as were patients with incomplete medical records. A total of 41 patients met these criteria, of which 11 ovarian CCC tumors and five uterine CCCs were selected for this exploratory analysis.

A HIPAA-exempt IRB approval was obtained (UCI IRB HS#2015-2464). Available clinical information included age, ethnicity, tumor origin, International Federation of Gynecology and Obstetrics (FIGO) tumor stage (1–4), date of diagnosis, and date of death or last documented encounter. These data were abstracted from the electronic medical record system as well as the University of California, Irvine, Tumor Registry. Overall survival was calculated in months by subtracting date of death from the date of original diagnosis. Censored survival time was used for living patients, utilizing the last documented follow-up exam date to calculate survival in months.

### Immunohistochemistry

All immunohistochemistry was done using the automated Ventana Medical Systems-Ultra Roche Tissue Diagnostics platform. For PD-L1, Ventana SP263 rabbit monoclonal antibody was used, and the results were reported as 0% (negative) or >0% (positive), based on institutional standards. As there is not an FDA-approved companion diagnostic anti-PD-L1 assay for gynecologic cancers at this time, the Ventana SP263 antibody was chosen as this was the institutional standard of care and data suggest that it is comparable to other clonal assays. We also calculated the PD-L1 combined positive score (CPS) as the number of PD-L1 positive cells (including tumor cells, lymphocytes and macrophages) divided by the total number of viable tumor cells, multiplied by 100. The CPS results were reported as <1 or ≥1 (16). Immunohistochemical expression of PD-L1 in ovarian and uterine CCC has been in explored in further detail in a larger cohort, from which this patient sample was selected (17).

### RNA Sequencing

Tissue blocks were evaluated by a pathologist to identify the most homogeneous area of CCC from which RNA could be extracted. The FFPE RNA/DNA Purification Plus Kit (Cat # 54300, Norgen Biotek Corp) was used to isolate total RNA from patient specimens. RNA quality and quantity were assessed using an Agilent 2100 Bioanalyzer and a Qubit fluorimeter, respectively. All samples exhibited a DV200 metric of >30% of RNA with fragment sizes >200 nucleotides, as described previously (18, 19).

The Illumina TruSeq RNA Access library preparation kit was used for gene expression profiling of the 11 ovarian and five uterine tumors, as described previously (18, 19). Publicly available raw RNA-seq data using TruSeq RNA Access library prep kit from 10 BRCA-mutated high grade serous ovarian cancers (HGSOC) (GSE141142) was collected and directly compared with our ovarian CCC RNA-seq data (20). The same methods of library preparation (*i.e.*, TruSeq RNA Access of Illumina) were used in both studies allowing for direct comparison of transcriptome between the two different histologic subtypes. The Access method of Illumina is based on 400,000 oligonucleotides (the Manifest) that are complementary to coding RNA. Sequencing reads that mapped to the Truseq RNA Access oligonucleotides were used for further analysis. A multiple comparison correction using a Benjamini–Hochberg FDR of 0.05 was used. Similarly, raw RNA-seq data was collected from 10 pairs of matched metastatic and primary HGS ovarian cancers, and seven pairs of matched primary HGS ovarian tumors and normal fallopian tubes (GSE137237) (21). We used oligo capture method (Illumina) to obtain sufficient RNAseq data from ovarian CCC samples, whereas this publicly available data used whole transcriptomes (Illumina). Thus, it was necessary to perform these comparisons indirectly although we applied the same alignment and normalization methods to our data and the external dataset. The transcriptome profile of PD-L1+ *versus* PD-L1− ovarian CCC was compared to 10 matched metastatic HGSOC with matched primary tumors, as well as seven primary HGSOC and matched normal fallopian tubes (GSE137237) (21).

### Statistical Analysis

Transcript quantification was performed using DESeq2 normalization (Strand NGS) followed by normalization to the median of all samples (22). Pooled analysis was performed using the Audic-Claverie Test (AC) test (23). The Benjamini–Hochberg correction was applied to account for multiple testing and a corrected p-value of 0.05 was used as the threshold for detection of differentially expressed genes. Molecular pathway and functional analyses of statistically significantly differentially expressed genes of multiple experiments were analyzed using the Ingenuity Pathway analysis (IPA) package (QIAGEN Inc, USA). Canonical pathways and diseases/functions were deemed overrepresented at –log_10_ (p-value) >1.3, which is p<0.05.

### Metadata Analysis of Ovarian Cancer RNA-Seq

For a meta-analysis of ovarian cancer RNA-seq data, we reanalyzed recently published GEO RNA-seq (GSE137237) data from seven matched pairs of primary HGS ovarian tumors and normal fallopian tubes, as well as ten matched pairs of metastatic and primary HGS ovarian cancer separately (21). Raw RNA-seq data were collected from the European Nucleotide Archive (ENA, https://www.ebi.ac.uk/ena) and imported into the Strand NGS tool to be analyzed at meta levels with our RNA-seq data from clear cell ovarian cancer patients.

### Identification of the Immune Microenvironment Using CIBERSORT

**Figure 1:**
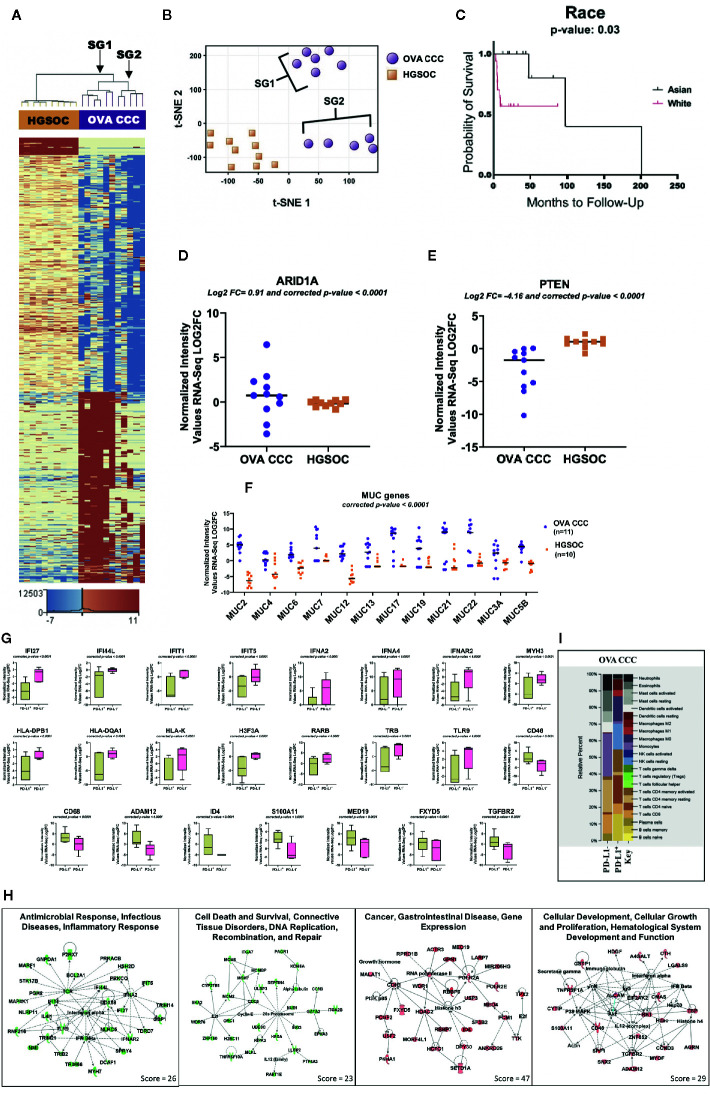
RNA-seq analysis of clear cell ovarian cancer. **(A)** Eleven clear cell ovarian carcinoma specimens were compared to ten high grade serous ovarian cancer patients (HGSOC). 1,212 genes were upregulated and 1,613 genes downregulated in ovarian CCCs compared to HGSOC (corrected p-value < 0.05, |FC| ≥ 10). Two distinct subgroups (SG1 and SG2) are revealed. **(B)** A non-linear dimension reduction (t-SNE) 2D plot on the 2,825 statistically differentially expressed genes in ovarian CCC and HGSOC revealed the same two distinct subgroups as in **(A)**. Orange squares are HGSOC. **(C)** Kaplan–Meier curve for 11 Asian and 19 White patients with ovarian CCC displaying the effects of race on ovarian cancer overall survival. **(D, E)** Log_2_ expression levels of tumor suppressor genes *ARID1A* and *PTEN* in eleven ovarian CCCs and ten HGSOC patients. **(F)** Scatter plot of the expression of mucin gene families. Mucin gene family were expressed at significantly higher levels (p < 0.0001) in ovarian CCC than in HGOC. **(G)** Box plots depicting the expression levels of significantly differentially transcribed genes (corrected p < 0.0001, |FC| ≥8) in PD-L1 CPS+ patients (n = 6) compared to PD-L1 CPS− patients (n = 5). **(H)** Four top ranked gene networks of up and downregulated genes in PD-L1+ as compared to PD-L1− ovarian CCC patients. Green colored nodes indicate downregulated and red indicates upregulated gene expression in PD-L1+ *versus* PD-L1− ovarian CCC cases. Darker shades of the nodes indicate higher expression values. Solid lines represent direct interactions. Dotted lines represent indirect interactions. White colored nodes are in the pathway, but not in our dataset. **(I)** CIBERSORT analysis of 22 immune cell types in PD-L1 positive (n = 6) and negative ovarian CCC (n = 5) cohorts (1,000 permutations). **(**OVA CCC, ovarian clear cell carcinoma; HGSOC, high grade serous ovarian cancer; FC, fold change; OS, overall survival; PD-L1, programmed death ligand 1; SG1, subgroup 1); SG2, subgroup 2.

**Figure 2:**
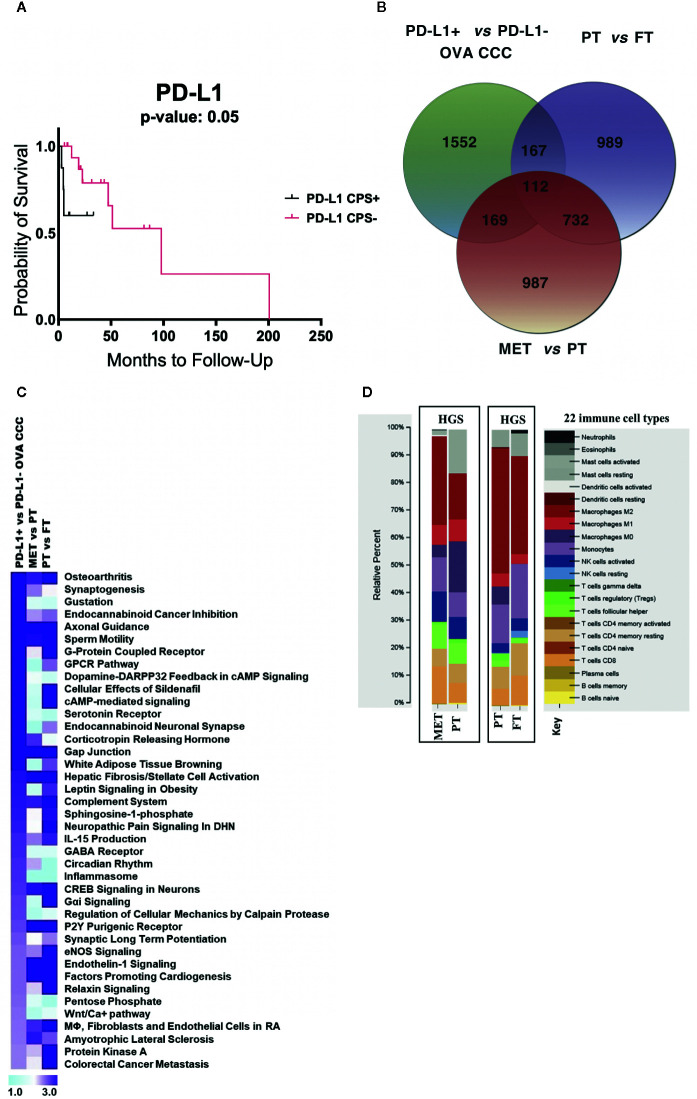
RNA-seq metadata analysis of ovarian cancer. **(A)** Kaplan–Meier curve displaying the effects of PD-L1 expression on ovarian cancer overall survival (11 PD-L1+ and 21 PD-L1−). **(B)** Venn diagram depicting the number of unique and overlapping differentially transcribed genes among top 2,000 genes across three different comparisons: (i) PD-L1+ (n = 6) *vs* PD-L1− (n = 5) CCC ovarian cancers; (ii) seven pairs of primary high grade serous ovarian cancers (HGSOC) and normal fallopian tubes; (iii) ten pairs of metastatic and primary HGSOC. **(C)** Comparative pathway analysis across the three datasets, sorted based on significantly enriched pathways (−log_10_ p-value > 1.9) in ovarian CCC compared to HGSOC patients. **(D)** CIBERSORT analysis of the relative proportions of 22 tumor infiltrating immune cell types (25) between HGSOC metastatic *versus* primary tumor, and between HGSOC primary tumors *versus* matched normal fallopian tubes (1,000 permutations). OVA CCC, ovarian clear cell carcinoma; HGSOC, high grade serous ovarian cancer; MET, metastatic tumor, high grade serous ovarian cancer; PT, primary tumor, high grade serous ovarian cancer; FT, normal fallopian tube.

**Figure 3:**
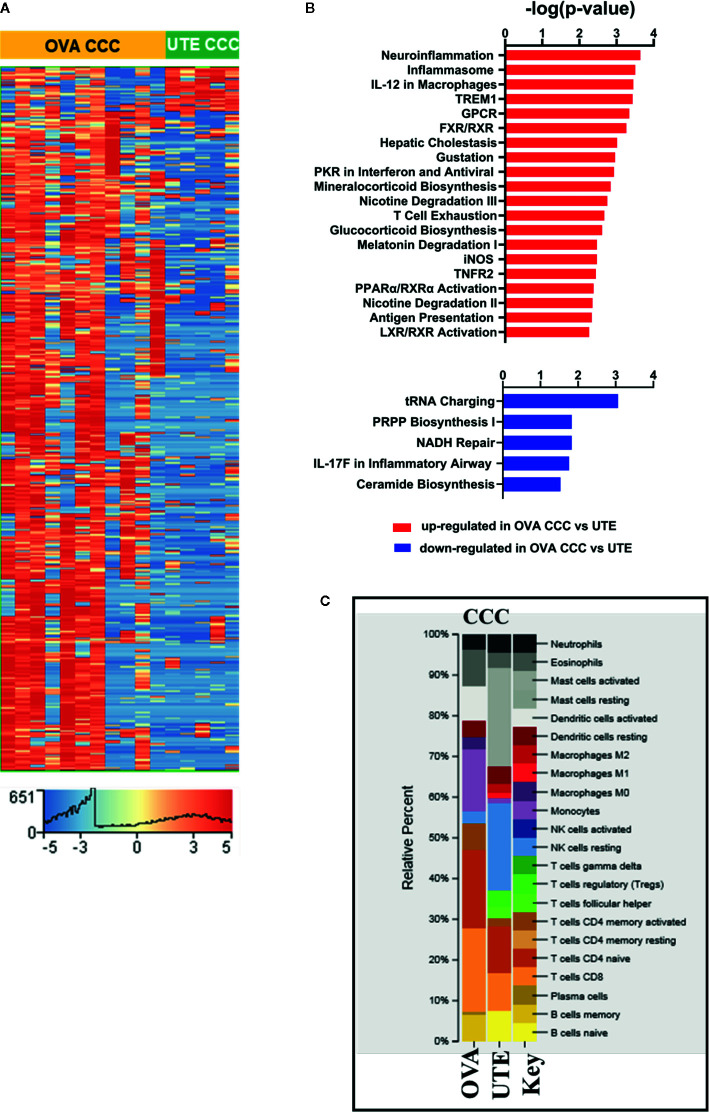
RNA-seq analysis of clear cell ovarian and clear cell uterine cancer. **(A)** Heat map showing the hierarchical clustering of 1,716 differentially expressed genes (corrected p < 0.05, |FC| ≥10) in clear cell ovarian (n = 11) *versus* clear cell uterine (n = 5) cancer. **(B)** Bar diagrams depicting the top 20 significant pathways (p < 0.05) associated with upregulated genes and top five significant pathways (p < 0.05) associated with downregulated genes in OVA CCC as compared to UTE CCC. **(C)** CIBERSORT analysis of immune cell compositions in clear cell ovarian and uterine carcinomas. The frequency of infiltrated immune cell types was estimated using gene expression profiling data of the two sites. OVA CCC and OVA ovarian clear cell carcinoma; UTE and UTE CCC, uterine clear cell carcinoma.

We used the computational tool “Cell-type Identification By Estimating Relative Subsets Of RNA Transcripts” (CIBERSORT) (24) to characterize the immune cell-type composition (22 immune cell types) of multiple cohorts with varying gene expression profiles (*i.e.* ovarian CCC versus uterine CCC, PD-L1+ *versus* PD-L1− ovarian CCC, HGS metastasis *versus* matched primary, and ovarian HGS primary tumor *versus* matched normal fallopian tube. We used normalized quantified values as input to CIBERSORT. In this exploratory analysis, we calculated the relative immune fraction score, which estimates the fraction of each immune cell type such that the sum of all fractions is equal to 1 (total leukocyte content) for a given mixture sample. Stacked bar graphs were generated by the CIBERSORT server **(** **Figures 1I**, **2D**, **3C** **)**.

## Results

### Patient Characteristics

Of the 11 patients in the ovarian CCC cohort, there were three at FIGO stage 1, three at stage 2, three at stage 3 and two at stage 4. Patients self-identified as Asian (4/11) or white (5/11), with 2/11 unidentified. Mean overall survival (OS) was 15.2 months (range 0–43). Within the five patients in the uterine CCC cohort, three were at FIGO stage 1, one at stage 3 and one at stage 4. Patients self-identified as white (2/5), or black (2/5), with 1/5 unidentified. Mean OS was 30.08 months (range 7.3–48.8). Patients’ characteristics and PD-L1 staining results are included in **Table S1**.

### RNA-Seq Analysis of Clear Cell and High Grade Serous Ovarian Carcinoma

A comparison of the transcription profiles of ovarian CCC to the more common high-grade serous ovarian carcinoma (HGSOC) histology is critical to developing targeted therapeutic strategies. This analysis identified 1,212 genes that were statistically significantly upregulated and 1,613 that were statistically significantly downregulated (FDR < 0.05 and |FC| ≥10) in clear cell ovarian *versus* HGSOC (**Figure 1A**, **Supplementary Dataset 1, ST1A**).

### Pathway Analysis of Differentially Expressed Genes in Ovarian CCCs Compared to HGSOC

The core pathway analysis in the IPA tool was used to identify the most significant pathways represented among the 1,613 downregulated and 1,212 upregulated genes in ovarian CCC (n = 11) compared to HGSOC (n = 10). Among the top canonical pathways associated with downregulated genes in ovarian CCC as compared to HGSOC are mitochondrial dysfunction, oxidative phosphorylation, protein ubiquitination, PI3K/AKT pathways, NER pathways, integrin signaling, BMP, ATM signaling, hypoxia signaling, and several immune response pathways including IL-2 and IL-17. Downregulated genes in ovarian CCC as compared to HGSOC include NDUFA1, NADUFA12, NDUFA13, NDUFAB1, NDUFB5, NDUFB9, NDUFS6, NDUFS8, UBE2B, UBE, PTEN, SUMO1, and CDC34 (**Supplementary Dataset 1, ST1B-C**). The significantly upregulated pathways in ovarian CCC as compared to HGSOC include multiple metabolic pathways (nicotine degradation, role of lipids, thyroid hormone metabolism, glucocorticoid receptor signaling, serotonin degradations, glycine, and retinoate biosynthesis) and immune response pathways (RIG1-like receptors in antiviral innate immunity, and the role of cytokines in mediating communication between immune cells) (**Supplementary Dataset 1, ST1C**)

### Subcategorization of Clear Cell Ovarian Cancer

RNA-seq analysis of the ovarian CCC samples revealed two potential distinct subgroups in a tSNE plot (**Figures 1A, B** **)**. This distinction was not observed with conventional pathology approaches (*i.e.* cell morphology). Subcategorization of clear cell cancers has not been previously described. The differences in gene expression in these two subgroups of CCC ovarian cancer patients dichotomized based on ethnicity. Subgroup 1 contained 66% white patients (4/6 white and 2/6 unknown), whereas subgroup 2 contained 80% women of Asian ancestry (4/5 Asian and 1/5 white). Using a larger ovarian CCC IHC-only cohort of 30 patients from which our pilot population was derived, Kaplan–Meier analysis of disease-specific overall survival suggests a trend that White women with ovarian CCC may have worse overall survival at a median 53.8 months compared to Asian American women at a median 4,101 months (p = 0.29) (**Figure 1C**) (6) (17).

We investigated the expression levels of genes that are well-established targets of somatic mutations within clear cell ovarian cancers as possible drivers of these two subgroups. Within the overall ovarian CCC cohort, *ARID1A* was expressed at significantly higher levels (corrected p-value < 0.05 and log_2_ FC = 0.91) and *PTEN* at significantly lower levels (log_2_ FC = −4.16) when compared to HGSOC (**Figures 1D, E** **).** Expression of *ARID1A* between the two ovarian CCC subgroups was significantly upregulated in subgroup 2 containing Asian American women, but not in subgroup 1 (log_2_ FC =2.78). In contrast, *PTEN* expression was significantly downregulated in both subgroups when compared to HGSOC (log_2_ FC = −4.12 in SG1 and log_2_ FC = −4.20 in SG2) (**Figure 1E**).

We found increased mRNA expression of several mucin genes in ovarian clear cell carcinoma patients (n = 11), when compared to HGSOC (n = 10), including MUC2, MUC4, MUC6, MUC7, MUC12, MUC13, MUC17, MUC19, MUC21. MUC22, and MUC5B (**Figure 1F** **).** Additional overexpressed genes were those encoding fork-head box transcriptional factor FOXA1 and FOXD4. Among significantly differentially under-expressed genes in ovarian CCC compared to HGSOC were F-box leucine-rich repeat proteins (FBXL2, FBXL13, FBXL3) and several cytochrome genes (COX15, COX17, COX18, COA6, SCO1, and SCO2) (**Supplementary Dataset 1, ST1A** **)**.

### Pathway Analysis of Ovarian CCCs With or Without Immunohistochemical PD-L1 Expression

Patients were divided into two groups, where PD-L1 combined positive scores (CPS) <1 was considered negative and PD-L1 CPS ≥1 was considered positive (26). Of the 11 investigated ovarian CCC samples, six were positive for PD-L1 expression and five were negative. The expression of 332 genes was positively correlated with PD-L1 expression, and 2,920 were negatively correlated (corrected p-value < 0.05 and |FC| ≥8) (**Supplementary Dataset 1, ST1D**). Among significantly negatively correlated genes with PD-L1 expression are genes of DNA repair (MYH3, MYH6, MYH7, and MYH13), antigen presentation and innate immune response (IFI27, IFI44L, IFIT1, IFIT5, IFNA4, DDX58, IFNA2, TLR9, TRB, and IFNAR2) (**Figures 1G, H**, **Supplementary Dataset 1, ST1D**). PD-L1 expression positively correlated with high expression levels of tumor promoting genes such as CD68, ADAM12, FXYD5, S100A11, CD46, and MED19 (**Figures 1G, H**, **Supplementary Dataset 1, and ST1D**).

To identify networks associated with the genes significantly up- or downregulated genes in PD-L1+ as compared to PD-L1− patients, we used the “core analysis” module in IPA (**Supplementary Dataset 1, ST1E-F**). Among the top networks negatively correlated with PD-L1 expression are (a) “Antimicrobial Response, Infectious Diseases, Inflammatory Response” and (b) “Cell Death and Survival, Connective Tissue Disorders, DNA Replication, Recombination, and Repair” **(** **Figure 1H**, **Supplementary Dataset 1, ST1E**). Two important hub genes that negatively correlated with PD-L1 expression are IFNA2 (interacting with IFI27, IFIT5, DDX58, TRIM14, IFI44L and HSH2D) and TCR (interacting with IFIT1, PGK2, IFI44L, BCL2A1, P2RX7, MARF1 and STK17B). Additionally, cell death and repair network including CDC6, MCM6, ITGA7, GATA2, PTP4A3 and SKA2 are negatively correlated with PD-L1 expression **(** **Figures 1G, H**, **Supplementary Dataset 1, and ST1E**).

Networks associated with genes positively correlated with PD-L1 include “Cancer, Gastrointestinal Disease, Gene Expression” and “Cellular Development, Cellular Growth and Proliferation, Hematological System Development and Function”). In the first network, Histone 3 (H3F3A) is a key hub protein, and the RNA of this gene is downregulated in PD-L1+ OVA CCC patients **(** **Figures 1G, H**, **Supplementary Dataset1, ST1D, and ST1F**). This gene has network interactions with several proteins whose transcripts are upregulated including HDAC2, ID4, SF3B, DPY30, USP3 and RNA polymerase 2 (**Figures 1G, H**, **Supplementary Dataset 1, ST1F**). The second network centered on several genes including CD46, TGFBR2, and ALCAM. These genes have network interactions with several proteins with higher mRNA expression levels in PD-L1 expressing cases, including ADAM12, MYOF, SPP1 and SNX2 (**Figures 1G, H**, **Supplementary Dataset 1, ST1F**).

### The Immune Microenvironment of Ovarian CCCs With and Without PD-L1 Expression

CIBERSORT was used to estimate the relative fractions of 22 tumor infiltrated leukocyte types in PD-L1− (n = 5) as compared to PD-L1+ (n = 6) clear cell ovarian cancer patients. Our data revealed higher average fractions of some immune system cell types in the five PD-L1− as compared to the six PD-L1+ CCC tumors. These included monocytes (26 *vs* 2%), resting memory CD4 T-cells (20 *vs* 2%), CD8 T− cells (11 *vs* 6%), eosinophil (12 *vs* 0.6%), and neutrophils (10 *vs* 2%), activated dendritic cells (6 *vs* 0%), activated mast cells (7 *vs* 0%), and plasma cells (4 *vs* 0%). In contrast, memory B cells (16%) and resting NK cells (15%) were present in PD-L1+ and completely absent in PD-L1− (**Figure 1I**).

### Metadata Analysis of RNA-Seq for Ovarian PDL-1+ and PD-L1− CCC and Non-Clear Cell Populations

Existing data suggests that PD-L1 expression may be associated with poor prognosis in several cancer types. Using a larger sample from our larger cohort of IHC-only patients (n = 32), a Kaplan–Meier curve for disease-specific survival in the ovarian CCC cohort suggests a trend that women with positive PD-L1 have worse overall survival (undefined), compared to PD-L1 negative women at a median OS of 101 months (p = 0.35) (6) (**Figure 2A** **).**

To compare the transcriptome profile of PD-L1 positive *versus* negative ovarian CCCs with metastatic HGS and primary HGSOCs, we collected publicly available raw RNA seq data from Mitra et al. study (GSE137237) (20). The top 2,000 statistically significantly differentially expressed genes (corrected p < 0.05) for various group comparisons were analyzed, including ovarian PD-L1+ *versus* PD-L1− CCC, HGS metastatic *versus* matched HGS primary tumors, and HGS primary tumor *versus* normal fallopian tubes (**Supplementary Dataset 2, ST2A-C**). These comparisons revealed 987 genes that were uniquely regulated when metastatic HGS tumors were compared with HGS primary tumors, 989 such genes when primary HGS tumors were compared to normal fallopian tubes, and 1,552 such genes when PD-L1 positive ovarian CCCs were compared to PD-L1 negative ovarian CCCs (**Figure 2B**, **Supplementary Dataset 2, ST2A−C** **)**. When those genes with differential expression in the PD-L1+ *versus* PD-L1 negative groups were compared with those in the HGS metastatic tumor *versus* HGS primary tumor groups, we identified 281 overlapping genes of which 206 (73%) were concordant (**Figure 2B**, **Table 1** **)**. Examples of upregulated concordant genes between PD-L1+ ovarian CCC and metastatic HGS include ARPC4, CD53, IGFN1, PSMD4, TGFBR2, TMSB10, and WDR1. Among downregulated concordant genes between both PD-L1+ ovarian CCC and metastatic HGS ovarian patients were WNT3A, WNT5A, and H3F3A. Pathway analysis of these concordant genes suggests alterations in the role of NANOG in mammalian embryonic stem cell pluripotency, aldosterone signaling in epithelial cells, STAT3 pathway, axonal guidance, and *PTEN* signaling (**Supplementary Dataset 2, ST2A–B, ST2D**). In addition, ovarian CCC shared 279 overlapping genes with primary HGS tumors, of which 179 were concordant genes (64%) (**Table 1**).

**Table 1:** Statistical evaluation of comparative gene expression.

	OVA CCC *vs* PD-L1+/−	MET *vs* PT	PT *vs* FT
***Patients***	6/5	10/10	7/7
***Regulated genes and direction of regulation***	204↑, 1,796↓	397↑, 1,603↓	897↑, 1,103↓
***Overlapping genes with OVA CCC PD-L1+/−***		281	279
***Concordant genes with OVA CCC PD-L1+/−***		7↑, 199↓	19↑, 160↓
***Dis-concordant genes with OVA CCC PD-L1+/−***		75	100

The number of up- (↑) and down- (↓) regulated genes with the same direction of regulation in each comparison is shown. OVA CCC, ovarian clear cell carcinoma; Met, metastatic high grade serous ovarian cancer; PT, primary high grade serous ovarian cancer; FT, normal fallopian tube.

Core canonical pathway analysis for the most significantly differentially expressed 2,000 genes in each dataset was used to identify 56 pathways that were enriched for differential gene transcription in PD-L1+ ovarian CCC as compared to PD-L1− ovarian CCC, 164 such pathways for metastatic HGS cancer compared to primary HGS tumors, and 201 such pathways for primary HGSOC compared to normal tubal tissue. Comparison of these data was performed across the three datasets then sorted according to the top 40 pathways enriched for differential gene transcription in the ovarian PD-L1+ *versus* PD-L1− cohorts of ovarian CCC (**Figure 2C**, **Supplementary Dataset 2, ST2E** **)**. Of the top 40 signaling pathways with −log_10_ (p-value) >1.9, nine showed enrichment exclusively in PD-L1+ clear cell ovarian cancer as compared to metastatic HGS and primary HGS ovarian cancer including circadian rhythm, several metabolic pathways such as pentose phosphate, WNT/Ca+ signaling and GABA receptor signaling. There were two signaling pathways corticotropin releasing hormone and synaptogenesis showing similar enrichment in both PD-L1+ clear cell and HGS metastatic ovarian cancer. Moreover, when considering ovarian PD-L1+ CCC and HGS primary ovarian cancers an additional 15 signaling pathways were identified with similar enrichment of differential gene transcription, including G*α*i and colorectal cancer metastasis **(** **Figure 2C**, **Supplementary Dataset 2, ST2E** **).**

### Identification of Leukocytes Infiltrating the Tumors of Non-Clear Cell Ovarian Cancer

CIBERSORT analysis was performed to compare the immune cell type composition in paired HGS metastatic versus primary ovarian tumors (n = 10) and paired HGS primary tumors *versus* normal fallopian tubes (n = 7). Metastatic HGS tumors showed higher fractions of M2 macrophages (32%) as compared to their matched primary tumors (17%). Conversely, activated mast cell and M0 macrophages represented 2 and 4% in metastatic HGS tumors, as compared to 16 and 19% in matched primary HGS tumors. When immune cell type composition in HGS primary tumors were compared to their matched normal fallopian tubes, both show high fractions of M2 macrophages at 45 and 35% respectively. There are higher proportions of resting mast cells (6 *vs* 8%) and resting CD4 memory T cells (8 *vs* 12%) in HGS primary tumors as compared to normal fallopian tubes respectively (**Figure 2D**).

### Transcriptome Analysis of Clear Cell Ovarian and Clear Cell Uterine Carcinomas

The RNA-seq data from 11 ovarian CCCs and five uterine CCCs were compared to detect differences based on tumor type. In clear cell ovarian *versus* clear cell uterine cancer, 1,607 genes were statistically significantly upregulated, and 109 genes were statistically significantly downregulated (FDR < 0.05, |FC| ≥10) (**Figure 3A**, **Supplementary Dataset 3, ST3A**). IPA analysis of these 1,607 upregulated genes revealed significant enrichment in several metabolic pathways (including mineralocorticoid biosynthesis, nicotine degradation and glucocorticoid biosynthesis) and cell signaling pathways (including TNFR2, LXR/RXR, and FXR/RXR activation, inflammasome, IL-12 signaling and production in macrophages, iNOS, T-cell exhaustion, activation of IRF by cytosolic pattern recognition receptors). Among signaling pathways associated with significantly downregulated genes in ovarian CCCs *versus* uterine CCCs are NADH repair, PRPP biosynthesis and IL17 in inflammatory airway (**Figure 3B**, **Supplementary Dataset 3, ST3B–C**).

We performed comparative pathways analysis of IPA to compare the top 2,000 significantly differentially expressed genes (corrected p-value <0.05 and |FC| ≥10) in late-stage (stages 3 and 4) and early-stage (stages 1 and 2) disease in both the ovarian CCC and uterine CCC cohorts (**Supplementary Dataset 3, ST3D-F**). Comparative pathway analysis identified pathways uniquely different in late-stage *versus* early-stage disease in ovarian CCC as compared to uterine CCC patients, including several metabolic pathways (D-myo-inositol (1,3,4)-trisphosphate biosynthesis, melatonin, nicotine,3-phosphoinositide degradations, tyrosine biosynthesis), as well as *PTEN* and interferon signaling, DNA double-strand break repair by homologous recombination. Additionally, there were unique pathways in Gap Junction, renin angiotensin, estrogen receptor, CCR5 signaling in macrophages, and Wnt/Ca+ pathway (**Supplementary Dataset 3, ST3F** **)**

Finally, CIBERSORT was used to compare the immune cell infiltration between the two clear cell cohorts (**Figure 3C**). Activated mast cells were increased in uterine CCC at 20%, naïve- B cells at 5%, T-reg at 6%, T cells follicular helper at 4%, and M2 macrophages at 4% while they were absent in ovarian CCC. The proportions of resting NK cells were increased in the uterine CCC cohort, while neutrophils, activated DCs, eosinophil, monocytes, CD8 T-cells and naïve CD4 T-cells were decreased, relative to ovarian CCC. The proportion of activated DCs and monocytes were significantly higher in ovarian CCC **(** **Figure 3C**).

## Discussion

Whole genome sequencing within serous ovarian cancers, endometrioid and carcinosarcoma uterine cancers of The Cancer Genome Atlas (TCGA) has identified clinically relevant mutations and epigenetic alterations that have applications in both prognosis and treatment. Similar data is largely missing in rare gynecologic cancer histologies. This study identified the unique transcriptomes of uterine and ovarian CCCs, using exploratory correlations with demographic factors and tumor characteristics.

Our RNA sequencing results are consistent with prior data that suggests numerous *MUC* genes are expressed at higher levels in ovarian cancers (**Figure 1F**) (27). Overexpression of the *MUC* genes is clinically relevant as mucins are large extracellular proteins allowing them to serve as biomarkers (indeed, MUC16 is also known as CA125), and their overexpression is well established as prognostic indicators (28). We found FOXA1 to be upregulated whereas COX15, COX17, FBXL2, and cytochrome genes (SCO1 and SCO2) were down regulated in ovarian CCC as compared to HGS tumors. Over expression of FOXA1 in ovarian cancer is consistent with existing literature, with aberrant expression implicated in tumorigenesis and invasive phenotype (29). Potential tumor suppressor activities of FBXL2 utilizing the ubiquitin-mediated degradation of crucial cell cycle regulators have been reported (30). Genes of cytochrome oxidase C also function as tumor suppressors, including low expression of SCO2, which in breast cancer populations is associated with poorer prognosis (31).

Our population showed downregulated genes in the ovarian CCC cohort as compared to the HGS ovarian cancers in several canonical pathways including the PI3K/AKT pathway, as well as in mitochondrial dysfunction, oxidative phosphorylation, protein ubiquitination, NER pathways, integrin signaling, BMP, and ATM signaling. The disproportionately high rate of mutation of PI3K/AKT in ovarian CCC has been established with a role in cell survival, proliferation and angiogenesis – indeed, therapeutic targeting with PI3K/AKT inhibitors and mTOR inhibitors has been investigated (32). The role of downregulation of ATM signaling in clear cell carcinomas is not reported, although high ATM protein and mRNA in HGS carcinomas correlates with poor survival and platinum resistance (33). There is compelling evidence suggesting the role of deregulation of mitochondrial, oxidative phosphorylation, and ubiquitination pathways in tumorigenesis (31, 34). There were numerous NADH dehydrogenase (uniquinone) 1 alpha (NDUFA) genes that were downregulated in our cohort relative to HGS cancers, which is in contrast to renal clear cell carcinoma in which NDUFA4L2 expression is high, serving as a novel molecular target for treatment (35). Suppression of NDUFA has been reported in tumorigenesis of basal cell carcinoma (36).

Key upregulated pathways in our ovarian CCC cohort as compared to HGS tumors included multiple metabolic pathways (nicotine degradation, role of lipids, thyroid hormone metabolism, glucocorticoid receptor signaling, serotonin degradations, glycine and retinoate biosynthesis) and immune response pathways (RIG1-like receptors in antiviral innate immunity, and the role of cytokines in mediating communication between immune cells). The upregulation of metabolic pathways in ovarian CCC cohorts as the predominant phenotypic expression of differentially expressed genes is consistent with the strong body of literature supporting that reprogramming of biosynthesis and continuous cellular proliferation are hallmarks of tumorigenesis (37). Indeed, metabolic reprogramming of glucose utilization is critical in tumor initiation and progression (38). Not unexpectedly, downregulated pathways in our data were primarily those involved in DNA damage response, which facilitate tumorigenesis through unregulated progression through the cell cycle and enable metabolic reprogramming (39). Two subtypes of renal cell carcinoma have been identified based on metabolic up- or downregulation of factors such as serotonin and glycine, both of which are upregulated in our population which is a novel observation (40).

Within the cohort of ovarian CCCs, heat maps of differentially expressed genes revealed two subgroups (**Figures 1A, B** **)**. Subcategorization of clear cell cancers has not been previously described. We focused on *ARID1A* and *PTEN* given their existing associations with CCCs. Transcription of the tumor suppressor gene *ARID1A* was significantly upregulated in subgroup 2, while tumor suppressor gene *PTEN* transcription was significantly downregulated in both subgroups as compared to HGSOC. A higher fraction of Asian women contributed to the cancer specimens in subgroup 2 compared to subgroup 1. Our study suggests that Asian women presenting with ovarian CCC have better overall survival (**Figure 1C** **).** This improved survival is similar to that noted in previously published Kaplan–Meier analyses by Fuh et al., who showed that compared to Caucasian women, Asian women were younger, had earlier stage cancers, and had improved 5-year disease specific survival (41).

Downregulation of DNA repair and innate immunity genes was notable in the PD-L1+ cohort, including MYH genes, IFI genes and IFNA genes. Upregulation of tumor promoting genes in the PD-L1 positive group were notable for CD68, MED19 and CD46 (**Figures 1G, H** **)**. Over expression of CD68 along with other tumor associated macrophages is associated with worse prognosis in hepatocellular carcinoma patients (42). PD-L1 expressing CD68 macrophage represent suppressor cell populations in ovarian cancer, which contribute to immune escape of ovarian cancer cells (43). MED19 in urothelial cancers promotes bone metastases and invasive behavior, and may serve as a therapeutic target (44). High CD46 expression may serve as a target for oncolytic viruses, with *in-vitro* studies showing promise with intraperitoneal application in ovarian cancer (45).

Our data highlighted two important gene hubs in PD-L1+ tumors, including IFNA2 and T-cell receptor (TCR) genes, with implications for downregulation of T-cell receptors and the antigen-presentation network (**Figures 1G, H** **)**. Data suggests that PD-L1 expression impacts ligand-induced T-cell receptor downregulation to prepare cytotoxic T-cells against their targets. This, in combination with decreased IFNA2, activated DCs, and altered antigen presentation enables an immune-tolerant environment that allows tumor to evade host recognition (46). Our findings were notable for a negative correlation between CDC6 and PD-L1 expression, suggesting that PD-L1+ patients that do not express CDC6 may have better prognosis (**Figure 1H**). High expression of CDC6 is associated with poor prognosis in HGSOC, as it activates and maintains checkpoint mechanisms in the cell cycle and may allow accelerated cellular proliferation—its negative correlation with PD-L1 expression may have implications in prognosis (47).

Several important genes emerged that negatively correlated with PD-L1 expression, including Histone 3 (H3F3A) (**Figures 1G, H** **)**. Histone 3F3A may be deposited at highly transcriptionally active regions of the genome, and although the impact of H3F3A in clear cell ovarian cancer is not understood, its mutation in glioblastoma and other tumors have been reported (48). Another gene network that was uniquely upregulated in PD-L1+ as compared to PD-L1− ovarian CCC included transforming growth factor (TGF*β*R2) and ALCAM (**Figures 1G, H** **)**. Signaling *via* the TGF*β* pathway is essential in cellular differentiation, apoptosis, invasion, angiogenesis and immune regulation and dysregulation in this pathway is established in ovarian cancer tumorigenesis and progression, revealing therapeutic targets that have been explored (49). Both transmembranous ALCAM and soluble ALCAM in ascites fluid have been investigated as possible tumoral biomarkers and may be indicators of an aggressive phenotype in ovarian cancer (50).

As data on the immune microenvironment is lacking in gynecologic populations, to date, investigation of other cancer types can be illuminating. Breast cancer is often characterized by high fractions of M0 macrophages, M1 macrophages, follicular type T cells and regulatory T cells, and low fractions of plasma cells (12). In breast cancer, immune signatures were independently predictive of disease-free survival (DFS) and overall survival (OS), with worse DFS and OS with higher cell fractions of M0 macrophages and high resting NK cell fractions (12). In a similar study within a lung adenocarcinoma cohort, poor prognosis was correlated with a lack of memory B cells, increased M0 macrophages, and high neutrophils, while follicular T helper cells were linked to more favorable outcomes (13). Within colorectal cancer populations, CIBERSORT data has been used to construct a diagnostic immune risk score as well as a prognostic immune risk score with more accuracy than traditional staging (15). Renal CCCs have particularly robust tumor-infiltrating immune cells (high Th17/Th2 cell ratio, high CD8+ T/Treg ratio), which are associated with improved survival (51). The correlation of an immune signature with patient outcomes to allow improved prognostication is novel in clear cell gynecologic cancers, and our data serves as an exploratory introduction to the unique clear cell immune microenvironment.

Data support that PD-L1 positivity may represent a more aggressive phenotype in ovarian clear cell cancers (52). Our analysis revealed an abundance of CD8 T-cells, monocytes, activated dendritic cells and eosinophils in PD-L1 negative ovarian CCCs compared to PD-L1 positive tumors (**Figure 1I**). In addition to CD8 T-cells, mature or activated DCs play a critical role in the generation of a clinically-favorable cytotoxic immune response in HGSOC microenvironment (53) (**Figure 1I** **).**

The microenvironment of endometrial cancers has recently been explored, with dysfunction of altered NK cells shown to be a key mediator in the lack of a cytotoxic anti-tumor response, facilitating tumor progression (54). The endometrial cancer microenvironment fosters a feedback loop of IL-6, aromatase, and *in-situ* estrogen elevations, which promotes tumorigenesis (55). No previous data exists for clear cell subtypes of endometrial cancer. Our CIBERSORT data shows that our sample of uterine CCC tumors were different from ovarian CCC. Uterine CCCs had higher proportions of activated mast cells, T-regs, resting NK cells and M2 macrophages and lower proportions of activated DCs suggesting less activated immune response in these patients as compared to ovarian CCC cohorts **(** **Figure 3C** **).**

Non-clear cell HGS ovarian cancers show a completely different immune cell type profile as compared to our sample of clear cell ovarian and uterine tumors (**Figures 1I**, **2D**, **3C**). CIBERSORT Analysis revealed evident differences in enrichment of immunosuppressive M2 tumor associated macrophages (TAM) between clear cell and non-clear cell ovarian cancers, with enrichment in metastatic HGS and primary HGS tumors, and complete absence in ovarian clear cell tumors. This is similar to results by Zhang et al., in which ovarian CCCs had lower density of M1 and M2 TAMs than HGS counterparts, which may impact prognosis (56). Interestingly, normal fallopian tube also showed evidence of an enriched M2 population (**Figure 2D**), which is consistent with a study by Ardighieri et al. showing an abundance of CD163+ M2 macrophages (57).

This exploratory transcriptome analysis is an important step in understanding the underlying biology of these rare tumors and has implications for biomarker development and future creation of novel therapeutics. One of the primary strengths of this study is the novel nature of the data, particularly the CIBERSORT immune composition analysis, and correlations of expression data with patient/tumor data and immunohistochemical expression data. The small sample size of this exploratory investigation is a limitation in all analyses, particularly when subsets of CCC are used. These transcriptome data and hypothesis-generating correlations will require further investigations with a larger sample size, as well as further exploration of functional analyses to provide verification and guide further clinical applicability. The intriguing correlation of subgroups with gene mutations and ethnicity highlights future areas of research.

## Data Availability Statement

The original contributions presented in the study are publicly available. This data can be found here: Gene Expression Omnibus, GSE160692.

## Ethics Statement

Ethical review and approval was not required for the study on human participants in accordance with the local legislation and institutional requirements. Written informed consent for participation was not required for this study in accordance with the national legislation and the institutional requirements. Written informed consent was not obtained from the individual(s) for the publication of any potentially identifiable images or data included in this article.

## Author Contributions

JA, LR, DM, and FR contributed to concept development and translational relevance, methodology development and interpretation, manuscript preparation and editing. ML assisted in data collection. DM and RE, clinical pathologist, reviewed the slides. FR performed RNA sequencing and data analysis as well as creation of figures, with assistance from GD and PR. AA provided expertise in immunology and MM provided expertise in cancer and molecular genetics, allowing improved methodology and manuscript revisions. All authors contributed to the article and approved the submitted version.

## Funding

This study was supported in part by institutional ACS grant 56/IRG-16-187-13, an institutional NIH T-32 training grant (Ruth L. Kirschstein NRSA Institutional Training Research Grant 2 T32 CA06039611), the Queen of Hearts Foundation as well as the Department of Pathology and Laboratory Medicine, University of California Irvine.

## Conflict of Interest

The authors declare that the research was conducted in the absence of any commercial or financial relationships that could be construed as a potential conflict of interest.
