# Supplementary material for: Transcriptome Analysis of Ovarian and Uterine Clear Cell Malignancies
Source: Front Oncol. 2020 Dec 22;10:598579. doi: 10.3389/fonc.2020.598579 (PMC7784081; doi:10.3389/fonc.2020.598579)
Supplement: Supplementary file 1 [file Table_1.docx]

## **Supplementary Materials**

Title: Transcriptome analysis of ovarian and uterine clear cell malignancies

Jill Alldredge^1^, Leslie Randall, Gabriela De Robles^2^, Anshu Agrawal^3^, Dan Mercola^2^, Marisa Liu^1^, Robert Edwards^2^, Pavneet Randhawa^1^, Michael McClelland^4^ and Farah Rahmatpanah^2^

**Supplementary Dataset 1. RNA sequencing analysis of ovarian clear cell carcinoma cohorts and high grade serous ovarian cancers**. RNA-seq was performed on 11 OVA CCC and compared to raw RNA-seq from 10 HGSOC cases obtained from publicly available data in the GEO database (GSE141142). We used the Audic-Claverie Test (AC), which pools read counts across samples and tests the differences between two pools based on an assumption that a Poisson distribution of counts applies (1,2). Multiple testing correction of Benjamini-Hochberg with an FDR of 0.05 and a maximum *p-value* of 0.05 was used. Gene expression differences that occur between PD-L1+ as compared to PD-L1- ovarian clear cell carcinoma cohort is shown, along with gene type, description, p-value, and corrected p-value/FDR. Multiple gene lists were subjected to Ingenuity pathway and network analysis. The list of pathways and networks enriched for differentially transcribed genes (p < 0.05), their ranked p-values, and the genes in our list of differentially expressed genes that encode proteins that participate in those specific pathways are shown.

Supplementary Table 1A: 2825 differentially transcribed genes in eleven clear cell ovarian cancer cases (OVA CCC) versus ten high grade serous ovarian cancer patients (HGSOC); corrected p < 0.05; |FC| ≥ 10.

Supplementary Table 1B: Canonical Pathways of 1613 downregulated differentially transcribed genes in eleven OVA CCC versus ten HGSOC; corrected p < 0.05.

Supplementary Table 1C: Canonical Pathways of 1212 upregulated significantly differentially transcribed genes in eleven OVA CCC versus ten HGSOC; corrected p < 0.05.

Supplementary Table 1D: 3252 differentially transcribed genes in six PD-L1 CPS+ clear cell ovarian carcinoma cases (OVA CCC) versus five PD-L1 CPS- OVA CCC; corrected p < 0.05; |FC| ≥ 8.

Supplementary Table 1E: Gene network analysis of 2920 downregulated genes in PD-L1+ (n=6) versus PD-L1- (n=5) OVA CCC patients; corrected p < 0.05; |FC| ≥ 8.

Supplementary Table 1F: Gene network analysis of 332 upregulated genes in PD-L1+ (n=6) versus PD-L1- (n=5) OVA CCC patients; corrected p < 0.05; |FC| ≥ 8.

**Supplementary Dataset 2. Metadata analysis of RNA-seq.** Publicly available raw RNA–seq data were collected from 7 primary HGS ovarian cancer with matched normal fallopian tubes and 10 matched metastatic and primary HGS ovarian cancers (GSE137237). RNA-seq reads were realigned to the hg19 UCSC reference genome (Strand NGS), reads were deduplicated and normalized using DESeq. The top 2000 genes with corrected p-values < 0.05 for PD-L1+ OVA CCC *vs* PD-L1- OVA CCC, matched MET HGS ovarian cancer *vs* matched PT HGS ovarian cancer and PT HGS ovarian cancer *vs* matched normal FT are shown, along with gene type, description, p-value, and corrected p-value. Ingenuity comparison pathway analysis was used to identify similarities and differences among the multiple comparisons. The list of pathways enriched for differentially transcribed genes (p < 0.05), their ranked p-values, and the genes in our list of differentially expressed genes that encode proteins that participate in those specific pathways are shown.

Supplementary Table 2A: 2000 most significantly differentially transcribed genes in six PD-L1+ clear cell ovarian cancer (OVA CCC) versus five PD-L1- OVA CCC; corrected p < 0.05.

Supplementary Table 2B: 2000 most significantly differentially transcribed genes in ten matched metastatic HGS ovarian cancers (MET) versus ten matched primary HGS ovarian cancers (PT); corrected p < 0.05.

Supplementary Table 2C: 2000 most significantly differentially transcribed genes in seven primary HGS ovarian cancers (PT) versus seven matched normal fallopian tubes (FT); corrected p < 0.05.

Supplementary Table 2D: Canonical pathways of 206 concordant genes between (ten matched MET HGS ovarian cancer versus ten matched PT HGS ovarian cancer) and (six PD-L1+ OVA CCC versus five PD-L1- OVA CCC); corrected p < 0.05.

Supplementary Table 2E: Comparative pathway analysis of 2000 most significantly differentially transcribed genes in (six PD-L1+ OVA CCC vs five PD-L1- OVA CCC), (ten matched MET HGS ovarian cancer vs ten PT HGS ovarian cancer), and (seven PT HGS ovarian cancer vs seven normal FT); corrected p < 0.05.

**Supplementary Dataset 3. Transcriptome analysis of ovarian and uterine clear cell carcinomas.** Gene expression analysis using *Strand NGS* was performed with 11 ovarian and 5 uterine clear cell cancers. The list of 1716 differentially expressed genes (corrected p-value < 0.05, FC ≥ ±4) in clear cell ovarian (n=11) *vs* clear cell uterine (n=5) cancers, along with their corrected p-values are shown. The list of pathways enriched for differentially transcribed genes (p < 0.05), their ranked p-values, and the genes in our list of differentially expressed genes that encode proteins that participate in those specific pathways are also shown. The top 2000 genes with corrected p-values < 0.05 are presented for ovarian CCC by late versus early stage and uterine CCC by late versus early stage. Identity and expression differences (log_2_ FC) of each gene, along with corrected p-values, are shown. Significant genes in late stage OVA and UTE CCC as compared to early stage were subjected to an Ingenuity pathway analysis. The top pathways enriched for differentially transcribed genes (p < 0.05) and the genes in our lists of differentially expressed genes that encode proteins that participate in those specific pathways are shown.

Supplementary Table 3A: 1716 differentially transcribed genes in eleven clear cell ovarian cancer cases (OVA CCC) versus five clear cell uterine cancer cases (UTE CCC); corrected p < 0.05; |FC| ≥ 10.

Supplementary Table 3B: Canonical pathways of 1607 up-regulated differentially transcribed genes in eleven OVA CCC versus five UTE CCC; corrected p < 0.05.

Supplementary Table 3C: Canonical pathways of 109 down-regulated differentially transcribed genes in eleven OVA CCC versus five UTE CCC; corrected p < 0.05.

Supplementary Table 3D: 2000 most significantly differentially transcribed genes in five late stage clear cell ovarian cancer cases (OVA CCC) versus six early stage OVA CCC; corrected p < 0.05.

Supplementary Table 3E: 2000 most significantly differentially transcribed genes in two late stage clear cell uterine cancer cases (UTE CCC) versus three early stage UTE CCC; corrected p < 0.05.

Supplementary Table 3F: Comparative pathway analysis of 2000 most significantly differentially transcribed genes in OVA CCC and UTE CCC between late and early stage; corrected p < 0.05.

**Table S1.** Metadata for ovarian clear cell carcinoma and uterine clear cell carcinoma. Patients’ race, PD-L1 expression data, disease stage, and RNA-seq statistics are shown. OVA, ovarian clear cell carcinoma; UTE, uterine clear cell carcinoma.

| Patients | Subgroup  (SG) | PD-L1 CPS | Tumor stage | Race | Total number of reads | Aligned to transcriptome (%) |
| --- | --- | --- | --- | --- | --- | --- |
| OVA1 | 2 | 30 | 2 | Asian | 20,396,334 | 9,672,896 (47.40%) |
| OVA2 | 1 | 30 | 3 | Unknown | 156,804,172 | 40,114,356 (25.60%) |
| OVA3 | 1 | <1 | 1 | White | 27,761,490 | 11,482,779 (41.40%) |
| OVA4 | 1 | 20 | 3 | Unknown | 48,627,972 | 19,437,622 (40.00%) |
| OVA5 | 2 | <1 | 4 | Asian | 100,303,170 | 50,732,666 (50.60%) |
| OVA6 | 2 | 20 | 2 | Asian | 84,378,734 | 34,445,255 (40.80%) |
| OVA7 | 2 | 2 | 3 | Asian | 23,729,558 | 8,301,297 (35.00%) |
| OVA8 | 1 | <1 | 2 | White | 22,875,004 | 4,462,588 (19.50%) |
| OVA9 | 2 | 8 | 1 | White | 13,916,210 | 2,212,281 (15.90%) |
| OVA10 | 1 | <1 | 4 | White | 21,945,350 | 9,713,153 (44.30%) |
| OVA11 | 1 | <1 | 1 | White | 106,900,230 | 24,351,714 (22.80%) |
| UTE1 |  | <1 | 4 | Black | 67,923,298 | 28,743,675 (42.30%) |
| UTE2 |  | 1 | 1 | White | 47,551,830 | 17,811,150 (37.50%) |
| UTE3 |  | 1 | 3 | Unknown | 19,054,306 | 5,122,986 (26.90%) |
| UTE4 |  | 50 | 1 | White | 4,452,682 | 1,576,726 (35.40%) |
| UTE5 |  | <1 | 1 | Black | 66,960,992 | 34,639,330 (51.70%) |

**References**

1. Rahmatpanah F, Agrawal S, Jaiswal N, Ngyuen HM, McClelland M, Agrawal A. Airway epithelial cells prime plasmacytoid dendritic cells to respond to pathogens via secretion of growth factors. Mucosal Immunol **2018** doi 10.1038/s41385-018-0097-1.

2. Rahmatpanah F, Agrawal S, Scarfone VM, Kapadia S, Mercola D, Agrawal A. Transcriptional Profiling of Age-Associated Gene Expression Changes in Human Circulatory CD1c+ Myeloid Dendritic Cell Subset. J Gerontol A Biol Sci Med Sci **2018** doi 10.1093/gerona/gly106.
